# 1735. Impact of Children Universal Pneumococcal Vaccination (PCV7v/PCV13v) on Pneumococcal Invasive Disease Hospitalizations in a Private General Hospital, Uruguay

**DOI:** 10.1093/ofid/ofad500.1566

**Published:** 2023-11-27

**Authors:** Mercecedes Sanchez-Varela, Marcelo Chirella, Adriana Varela, Gabriela Algorta, Jorge Facal, María Catalina Pírez

**Affiliations:** British Hospital, Montevideo Uruguay, Montevideo, Montevideo, Uruguay; Hospital Britanico, Montevideo, Montevideo, Uruguay; British Hospital, Montevideo Uruguay, Montevideo, Montevideo, Uruguay; British Hospital, Montevideo, Montevideo, Uruguay; British Hospital, Montevideo Uruguay, Montevideo, Montevideo, Uruguay; Faculty of Medicin, University of the Republic, Montevideo, Montevideo, Uruguay

## Abstract

**Background:**

In 2008 Uruguay included pneumococcal conjugate vaccine (PCV) 7valent in children´s vaccination program (2+1). In 2010 PCV13 replaced PCV7. Since 2015, adults at risk of invasive pneumococcal disease (IPD) may receive 23 valent polysaccharide vaccine (PV23) or combined schedule PCV13+PV23.

Objective: to assess the impact of this strategy on IPD hospitalization.

**Methods:**

Rates per 10,000 discharges (Confidence Interval 95 %), diseases and serotypes (ST) are described before PCV7 vaccination (2006-2007), the year of vaccine implementation (2008), early after implementation (2009-2015) and late after implementation (2016-2022), the last 3 years during COVID-19 pandemic.

**Results:**

In 2006-2022 were 33 IPD cases in children and 88 in adults over 15 years old. Children´s hospitalization median annual rates were as follow 2006-2007: 50 (25-75); 2008: 18,5 (-2-39); 2009-2015: 8,5 (3-14); 2016-2022: 4 (0-8); percentages of rate reduction were 82,5% and 92% (p< 0.0000). In adults 2006-2007: 17 (10-25); 2008: 13,4 (4,62-22,1); 2009-2015: 6,6 (4,4-9); 2016-2022: 6 (3,5-8,5); percentages of rate reduction were 63,5% and 66 % (p= 0,0001).

Pneumococcal pneumonia (PP) was the most frequent disease in children 22/33 (66,6%), followed by bacteremia 8/32. PP was also in adults 68/88 (77.2,7%), followed by meningitis (7/88). One non vaccinated child died in 2008. Case fatality rate in adults was 9% (8/88), 3 died in 2006-2007, 2 in 2009-20215 and 3 in 2016-2022.

24 different ST were isolated in adults. Most frequent ST were 12F(15), 7F(11), 1(8), 8(7), 3(6) and 5(6). PCV13 would cover 38/88 (43%). A significant decline in PCV13 serotypes (PCV13-ST) was observed comparing the period prior PCV 17/22 (77%) with the late period 8/24 (34%) (p< 0.0000).

15 different ST were isolated in children: 26 PCV13-ST. 16 prior 2008: most frequent ST 1(5), 14(4), 3(2) and 5(2). In 2009-2022 13 hospitalizations; PCV13-ST: 1(2), 19A(2), 14(1), 6B< (1), 9V(1), 3(1) and non PCV13-ST: 7B/C(1), 24F(1), 13(1), 15A(1), 15B(1). A significant decline of PCV13-ST was observed comparing the period prior PCV (15/16, 94%) with the late period (2/4, 50 %) (p< 0.0000).

**Conclusion:**

There was a reduction in children’s discharge rates and in adults´ probably due to herd effect. Adult vaccination will improve individual immunization.

**Disclosures:**

**María Catalina Pírez, Pediatrician, Pediatric infectologist, microbiologist Professor of pediatric, Degree V**, Merck, Pfizer: Expert Testimony|Merck, Pfizer: Honoraria

